# Interplay of Amygdala and Cingulate Plasticity in Emotional Fear

**DOI:** 10.1155/2011/813749

**Published:** 2011-09-07

**Authors:** Hiroki Toyoda, Xiang-Yao Li, Long-Jun Wu, Ming-Gao Zhao, Giannina Descalzi, Tao Chen, Kohei Koga, Min Zhuo

**Affiliations:** ^1^Department of Physiology, Faculty of Medicine, University of Toronto, Medical Science Building, Room no. 3342, 1 King's College Circle, Toronto, ON, Canada M5S 1A8; ^2^Department of Neuroscience and Oral Physiology, Osaka University Graduate School of Dentistry, Suita, Osaka 565-0871, Japan; ^3^Department of Pharmacology, Fourth Military Medical University, Xi'an 710032, China; ^4^Department of Brain and Cognitive Sciences, College of Natural Sciences, Seoul National University, Seoul 151-747, Republic of Korea

## Abstract

The amygdala is known to be a critical brain region for emotional fear. It is believed that synaptic plasticity within the amygdala is the cellular basis of fear memory. Recent studies demonstrate that cortical areas such as the prefrontal cortex (PFC) and anterior cingulate cortex (ACC) may also contribute to the formation of fear memory, including trace fear memory and remote fear memory. At synaptic level, fear conditioning also triggers plastic changes within the cortical areas immediately after the condition. These results raise the possibility that certain forms of synaptic plasticity may occur within the cortex while synaptic potentiation takes place within synapses in the hippocampus and amygdala. This hypothesis is supported by electrophysiological evidence obtained from freely moving animals that neurons in the hippocampus/amygdala fire synchronous activities with cortical neurons during the learning. To study fear-related synaptic plasticity in the cortex and its functional connectivity with neurons in the amygdala and hippocampus will help us understand brain mechanisms of fear and improve clinical treatment of emotional disorders in patients.

## 1. Introduction 

**Figure 1:**
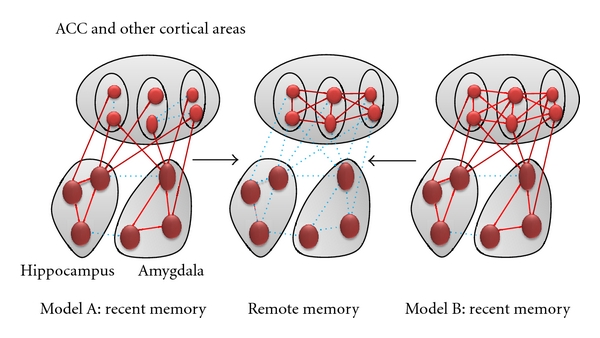
Interplay of the cortex and hippocampus/amygdala in fear memory. There are two major hypotheses related to the brain network involved in fear memory. Depending on the different types of conditioning protocols, it is likely that both mechanisms may take place. In model A, early fear memory is formed within the hippocampus and/or amygdala. At some time point after learning (e.g., during sleep), some of this information is replayed and transferred into the cortical synapses. After the formation of remote memory in the cortex, early synaptic changes are unlikely important. The exact synaptic and molecular mechanisms for the replaying remain to be investigated or proved. According to this model, the cortical activity is not required for the formation of early fear memory. In model B, early synaptic potentiation related to fear conditioning happens at synapses located in all three major areas, including the hippocampus, amygdale, and cortex. The interconnections among these three areas may be further enforced after the formation of early memory. Similar to model A, late or remote memory is mainly stored in the cortical synapses.

Fear is an adaptive response to pain or the threat of danger. It is believed that amygdala is a key brain area for fear. As the major cellular model used for understanding this neural mechanism, long-term potentiation (LTP), a type of long-lasting synaptic plasticity, has been predominantly studied in the amygdala. Consequently, much evidence suggests that LTP is required for the establishment and consolidation of fear memory [1]. In addition, the anterior cingulate cortex (ACC) is known as a key structure that contributes to not only the *recall* of fear memory [2], but also the *formation* of fear memory [3]. It has been elucidated how recent and remote fear memories are organized in the brain (Figure 1). To date, considerable evidence indicates that fear learning and memory are mediated by changes in synaptic strength in the ACC [4]. 

With the development of integrative approaches, including pharmacological and genetic manipulations, the molecular and cellular mechanisms underlying fear learning and synaptic plasticity in the amygdala and ACC have been elucidated. These range from membrane receptors, to intracellular signaling proteins, to transcription factors [4, 5]. The ACC is located between limbic and cortical structures to integrate emotion and cognition, thereby influencing amygdala-dependent learning [6]. Indeed, human and animal studies showed that neuronal activity in the amygdala and ACC changed when subjects were shown threatening faces or confronted with frightening situations, respectively [7, 8]. Thus, the functional connection between the amygdala and ACC may play an important role in fear learning and emotional processing. In this paper, we will first discuss recent evidence suggesting that fear learning and memory are mediated by changes in synaptic strength in the amygdala and ACC. We then discuss animal studies of molecular and cellular mechanisms underlying synaptic plasticity in the amygdala and ACC, which relate to fear learning and memory. Finally, we discuss anatomical and functional connectivity between the amygdala and ACC.

## 2. LTP Is a Cellular Model of Fear Learning

The brain undergoes plastic changes in response to peripheral stimuli and new experiences. Synaptic plasticity is a phenomenon referring to the ability of synapses to undergo long-lasting modifications after certain stimulation. LTP is one of the cellular mechanisms for various memories, such as spatial memory, fear memory, and chronic pain. Indeed, LTP shares many common features with long-term memory: they are both triggered rapidly by vigorous stimulations, have properties of associativity, may depend upon the synthesis of new proteins, and can last for significant amounts of time [9]. 

Fear conditioning is a well-established experimental model of fear learning, in which neutral conditioned stimuli (CS) are paired with aversive unconditioned stimuli (US) during behavioral training. The auditory (CS input) and somatosensory (US input) information can converge to the lateral amygdala through auditory thalamus-amygdala and auditory-cortex-(cortical-) amygdala pathways [10]. The acquisition of fear memory on auditory stimulation seems to be mediated by synaptic enhancements in the CS pathway, including both thalamic and cortical pathways [11–13]. Previous studies consistently indicate that LTP is the most likely synaptic mechanism underlying fear memory in the amygdala. First, electrophysiological studies using *in vitro* amygdala slices or *in vivo* recordings showed that auditory afferent pathways, including thalamic-amygdala and cortical-amygdala pathways, undergo synaptic potentiation after LTP-inducing stimuli [11, 12]. Second, the associative nature of LTP in the amygdala supports the notion that fear conditioning requires the convergence of odor and nociceptive inputs onto single neurons in the lateral amygdala [14]. Third, neural activity in the lateral amygdala has been shown to be modified during auditory fear conditioning in a manner similar to that observed after artificial LTP induction [15, 16]. Fourth, drugs that inhibit the induction and/or maintenance of LTP in the amygdala also inhibit fear memory [17]. Finally, fear conditioning occluded LTP-induced presynaptic enhancement of synaptic transmission in the cortical pathway to the lateral amygdala [12]. Therefore, synaptic transmission undergoes long-term plastic changes after training in the amygdala and may underlie processes involved in new information learning and storage within amygdala circuits.

## 3. Molecular Mechanism for the Induction of LTP in the Amygdala

Synaptic mechanisms for induction of LTP have been intensively investigated in amygdala slices. We describe mainly the molecular mechanisms for the induction of thalamic-amygdala LTP in the lateral amygdala. Induction of LTP in lateral amygdala neurons involves postsynaptic depolarization, which can cause the influx of Ca^2+^ from both NMDA receptors and L-type voltage-dependent Ca^2+^ channels (L-VDCCs) [12, 18]. Previous studies dissecting the role of GLUN2 (formerly NR2, see [19]) subunits in synaptic plasticity suggest that both the GLUN2A (NR2A) and GLUN2B (NR2B) subunits are involved in the induction of LTP in the lateral amygdala [20]. In addition, the phosphorylation of GLUN2B subunits is essential for synaptic plasticity in the amygdala and amygdala-dependent fear learning [21]. Another molecule involved in the induction of LTP is kainate (KA) receptors, which contribute to synaptic transmission in the amygdala [22]. We found that the deletion of GluK1 (GluR5) did not affect synaptic potentiation in the lateral amygdala, whereas in GluK2 (GluR6) knock-out mice, LTP induced by theta burst stimulation or pairing of synaptic activity with postsynaptic depolarization was blocked [22]. Recently, it is reported that the presynaptic GluK1 is involved in the induction of LTP in the lateral amygdala [23]. Furthermore, activation of metabotropic glutamate receptor (mGluR) subtype 5 is required for the induction of thalamic-amygdala LTP [24]. The involvement of protein kinases such as CaMKII [25] and CaMKIV [26] in amygdala LTP has also been implicated. Various forms of LTP are reported in the amygdala, depending on the presynaptic activity levels and degree of postsynaptic depolarization [11, 12, 27]. 

## 4. Molecular Mechanism for the Expression and Maintenance of LTP

After induction of LTP, the phosphorylation of AMPA receptors by the activity of CaMKII may mediate an increase in the number of AMPA receptors at synapses via activity-dependent AMPA receptor trafficking. In the lateral amygdala, synaptic delivery of the GluA1 (GluR1) subunit from extrasynaptic sites is the key mechanism underlying synaptic plasticity [28, 29]. Rumpel et al. found that in GluA1 knock-out mice, LTP at thalamic-amygdala pathway was completely abolished and that both auditory and contextual fear conditioning were impaired [28]. It is well established that the maintenance phase of LTP requires new protein synthesis and gene transcription [30, 31]. In particular, signaling molecules such as PKA, mitogen-activated protein kinase (MAPK), Ca^2+^/CaMKII, Egr-1 zinc finger transcription factor, PKM*ζ*, and fragile X mental retardation protein (FMRP) have also been implicated in the maintenance of LTP and consolidation of fear memory in the amygdala [32–35]. Of these, much attention has been paid to the role of PKM*ζ* in synaptic plasticity, as this kinase is the only molecule that is necessary and sufficient for maintaining LTP [36]. It is likely that PKM*ζ* maintains long-term memory by regulating GluA2-(GluR2-) subunit-mediated trafficking to the synapses in the amygdala [34]. Subsequently, these molecules activate the key transcription factor cyclic AMP-response element binding protein (CREB) as well as Egr-1 [37]. Phosphorylation of transcription factors including CREB activates nuclear transcription of relevant genes, thereby increasing protein synthesis [37]. These plasticity-related proteins are transported to specific synapses targeted for potentiation, which are required for the stabilization of LTP and long-term memory. 

**Figure 2:**
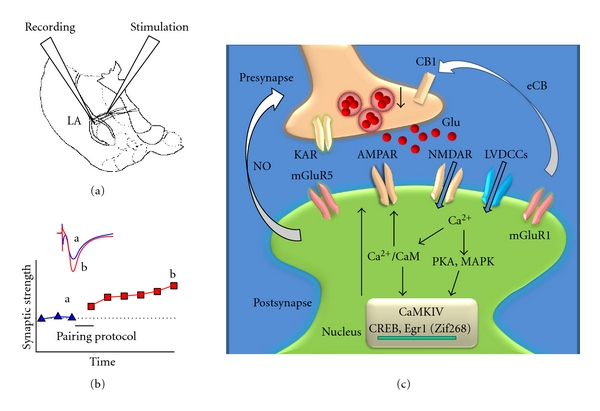
A cellular model for LTP in the lateral amygdale. (a) Diagram of an amygdala slice showing the placement of whole-cell patch-clamp recording and stimulation electrode. (b) LTP is induced by 80 pulses at 2 Hz with postsynaptic holding at +30 mV. (c) Activations of postsynaptic glutamate NMDA receptors and L-VDCCs lead to an increase in postsynaptic Ca^2+^ in dendritic spines. Ca^2+^ binds to CaM and leads to activation of Ca^2+^/CaM-dependent protein kinases (PKA, CaMKII, and CaMKIV). Subsequently, AMPA receptor will undergo plastic upregulation. Activation of CaMKIV, a kinase predominantly expressed in the nuclei, will trigger CREB signaling pathways. MAPK could translocate from the cytosol to the nucleus and then regulate CREB activity. mGluR5 and presynaptic KA receptors are involved in induction of LTP. Induction of thalamic LTP suppresses the presynaptic LTP by activation of presynaptic CB1 receptors by eCB released in response to activation of mGluR1.

In the amygdala, both presynaptic and postsynaptic mechanisms have been proposed for the expression of LTP; at thalamic-amygdala synapses, LTP is predominantly expressed postsynaptically, whereas at cortical-amygdala synapses, cAMP/PKA signaling is required for a presynaptic expression mechanism [11]. In addition, it has been reported that production of nitric oxide, a retrograde messenger, contributes to the expression of thalamic-amygdala LTP [38]. Interestingly, a recent finding suggests that the induction of thalamic LTP suppresses the presynaptic LTP coexisting at the same synapses; this mechanism is mediated by activation of presynaptic cannabinoid type 1 (CB1) receptors by endogenous cannabinoids (eCB) released in response to activation of the mGluR1 [23]. Molecular mechanisms underlying LTP in the lateral amygdala are shown in Figure 2.

## 5. Human Imaging Data of Fear Memory in the Amygdala

The neural correlates of fear memory in the human brain are increasingly being identified through neuroimaging studies. Several human observations have provided evidence for the involvement of the amygdala in fear learning [7, 39–42]. For example, a recent study combined functional magnetic resonance imaging (fMRI) with cued fear conditioning to investigate brain activity related to acquisition [41] and observed that tasks pairing visual cues with brief electrical shocks to the forearm induced corresponding activity within the amygdala. Similarly, fMRI observations have also identified activity within the amygdala in response to contextual fear conditioning, where subjects learn that a specific context is predictive of an aversive outcome [39–41]. More recently, an fMRI study found that predictable threat induced transient and sustained activity within the amygdala and ACC [43]. Correspondingly, observations of patients with amygdala damage have also suggested a critical role for this structure in fear learning. In one case, a patient with complete bilateral amygdala damage has been found to have severe deficits in the acquisition of auditory or visual cued fear conditioning [44]. Interestingly, this patient also presents an inability to detect fearful emotional expressions, suggesting an impairment in the ability to evaluate fear-related cues.

## 6. Evidence for the ACC in Fear Memory: The Animal and Human Imaging Studies

Although the amygdala has long been considered the emotional centre of the human brain [45], the ACC has robustly emerged as a critical component of a fear-processing network. The ACC is involved in the processing of pain-, emotion-, and threat-related stimuli [4]. Human electrophysiological recordings from ACC neurons have shown activity in response to noxious stimuli [46], and several human observations have underscored a necessary role of the ACC in pain affect [47]. During fear learning, both the amygdala and the ACC are activated, where activity within the amygdala may signal danger whilst the ACC establishes necessary neural activity to sustain attention to the threat [48]. Indeed in fear conditioning paradigms that introduce a time interval between a visual cue and shock [49], ACC activity has been found to decrease between the cue and shock presentations. This may represent a “coincidence detection” mechanism, whereby ACC activity during the visual cue and during the shock may signal that the two are indeed *related*.

Animal studies have also identified the ACC as a critical area involved in the acquisition and storage of fear memory. For example, the expression of pCREB in the ACC was significantly increased by auditory fear conditioning [26]. Consistently, the c-*fos* mRNA expression in the ACC increases by 50% by trace fear conditioning [50]. Infusion of excitotoxin (NMDA) into the ACC yielded to reducing freezing in trace-fear-conditioned mice [50]. Additionally, blocking of GLUN2B activities in the ACC by pharmacological or small interfering RNA could impair early memory of contextual fear [51]. Furthermore, experiments showed that electrical stimulation of the ACC induced fear memory [3]. These results suggest that the ACC is involved in the acquisition of fear memory. 

The contributions of the ACC to remote fear memory have been evaluated using contextual fear conditioning. Frankland et al. found that the recall of remote (36 days after conditioning) but not recent contextual fear memory (1 day after conditioning) elevated the expression of *Zif268* and c-*fos* in the ACC and that lidocaine infusion into the ACC disrupted remote fear memory [52]. The formation of remote fear memory is accompanied with neuronal structural change in the ACC [53]. These data suggest that the ACC also has a critical role for the storage of remote fear memory. 

## 7. Synaptic Plasticity in the ACC

The synaptic mechanisms underlying LTP have been elucidated in ACC slices. We here describe the molecular mechanisms for the induction and expression of LTP at layer V to layer II/III synapses in the ACC. Similar to the amygdala, the LTP induction in the ACC also needs the postsynaptic elevation of Ca^2+^, which was mainly mediated by NMDA receptors or L-VDCCs. The binding of Ca^2+^ to calmodulin (CaM) leads to the activation of calcium-stimulated signaling pathways. Mutants of a calcium-binding site in the N-terminal of CaM completely abolished the induction of LTP and LTD in the ACC [54]. In turn, Ca^2+^/CaM can stimulate the activities of adenylyl cyclases (ACs), which can convert ATP to cAMP. Among more than ten subunits of ACs in the CNS, AC1 and AC8 are two AC subtypes that respond positively to Ca^2+^/CaM [55]. Experiments using genetic and pharmacological approaches showed that AC1 is critical for the induction of LTP in the ACC [56, 57]. The increased cAMP binds to the regulatory subunit of PKA, which leads to the release of catalytic subunit of PKA. PKA could activate MAPK [58] or CREB, respectively. Subsequently, the activated MAPK likely has multiple targets including CREB that is required for long-term synaptic changes in ACC neurons. Ca^2+^/CaM can also activate different forms of CaM kinases; among them, CAMKIV is distinguished in its capacity to activate CREB-dependent transcription [59]. The role of CAMKIV in LTP has been identified by using *CAMKIV* gene KO or overexpression mice. It was found that ACC LTP was reduced or abolished by the deletion of the *CAMKIV* gene [26], while overexpression of CAMKIV enhanced LTP in transgenic mice [60]. A more recent study showed that the CAMKIV-CREB pathway is involved in translation-dependent early synaptic potentiation in the ACC [61]. 

**Figure 3:**
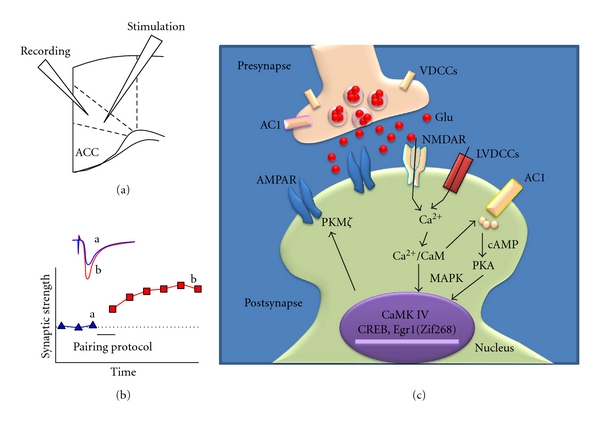
A cellular model for ACC plasticity. (a) Diagram of an ACC slice showing the placement of whole-cell patch recording and stimulation electrode. (b) LTP is induced by 80 pulses at 2 Hz with postsynaptic holding at +30 mV. (c) Activations of postsynaptic glutamate NMDA receptors or L-VDCCs lead to an increase in postsynaptic Ca^2+^ in dendritic spines. Ca^2+^ binds to CaM and leads to activation of calcium-stimulated ACs, mainly AC1 and other Ca^2+^/CaM-dependent protein kinases (PKC, CaMKII, and CaMKIV). Subsequently, GluA1-containing AMPA receptor will undergo the upregulation. Activation of CaMKIV, a kinase predominantly expressed in the nuclei, will trigger CREB signaling pathways. In addition, activation of AC1 leads to activation of PKA, and subsequently CREB as well. MAPK/ERK could translocate from the cytosol to the nucleus and then regulate the CREB activity. Postsynaptic PKM*ζ* is critical for maintaining synaptic potentiation in the ACC.

 Synaptic delivery of the GluA1 subunit from extrasynaptic sites is the key mechanism underlying synaptic plasticity [62]. In the ACC, LTP was abolished in the GluA1 KO mice [63]. Loading of GluA1 subunit C-terminal peptide analog (Pep1-TGL) into the recording electrode blocked the induction of cingulate LTP [64]. Thus, the interaction between the C-terminus of GluA1 and PDZ domain proteins is required for the LTP induction in the ACC. Furthermore, bath application of PhTx-433 five minutes after paired training reduced synaptic potentiation [64]. Therefore, our data suggest that Ca^2+^-permeable GluA2-lacking receptors contribute to the expression of LTP. The connection between PKM*ζ* and the maintenance of long-term memory has been demonstrated [65]. PKM*ζ* is a critical molecular player in the maintenance of late-phase LTP in the ACC [66]. Blocking the activities of PKM*ζ* by ZIP (*ζ*-pseudosubstrate inhibitory peptide) erased late-phase LTP induced by theta burst stimulation under field recordings [66]. Although there is no direct evidence to show which subtype of AMPA receptor was involved in the interaction of PKM*ζ* in the ACC, our results from neuropathic pain mice found that blocking the activities of PKM*ζ* decreased the expression of GluA1 in the synapses [66], which suggest that there is a tight connection between PKM*ζ* and GluA1 AMPA subunit in the ACC. Therefore, it is possible that PKM*ζ* is involved in the expression of cingulate LTP by interacting with GluA1. A model for molecular mechanisms underlying LTP in the ACC is shown in Figure 3.

## 8. Anatomic Connections between the ACC and Amygdala

The anatomic connections between the ACC and amygdala have been reported previously [67]. ACC neurons are directly projected to the amygdala, which has been confirmed in monkeys [68], cats [69], rabbits [70], and rats [71]. The topographic pattern from amygdala neurons to the ACC has been clarified by using anatomical tracing techniques, including horseradish peroxidase (HRP) or fast blue (FB) injections into the basolateral amygdala in rats. Most of the retrograde labeled neurons are found in the layers II, III, and V in the ipsilateral part of the ACC [71]. However, the majority of the labeled neurons in the contralateral ACC are seen in layers V/VI, with scattered neurons distributed in shallow layers [67]. Meanwhile, after wheat germ agglutinin (WGA) -HRP or injection of phaseolus vulgaris-leucoagglutinin (PHA-L) into the ACC, anterograde-labeled fibers were observed on both sides of the amygdaloid complex, but restricted to the medial part of the basolateral, basomedial, and lateral nucleus [67, 70]; no labeled fibers were observed in the central nucleus of the amygdala [70]. Amygdala neurons projecting to the ACC have also been observed in monkeys [72], cats [73], and rats [74]. Following injections of HRP or fluorescent tracers into the ACC, labeled neurons were mainly found in the medial part of the basolateral nuclei of the amygdala, although the basomedial, lateral, or basal accessory parts are also reported to obtain retrograde-labeled cells [74]. Besides the direct linkage, indirect linkage (ACC-thalamus-amygdala) makes up a very important circuit between the ACC and amygdala, as both anatomical and functional connections between the medial thalamus and ACC and those between the medial thalamus and amygdala connections have been observed [75]. 

## 9. The Network between the ACC and Amygdala during Fear Learning and Pain

Recent evidence suggests that the functional connection between the ACC and amygdala plays essential roles in fear learning and emotional processing including pain. Previously it was found that lesions of the basolateral amygdala blocked the memory-enhancing effect of posttraining intra-ACC infusions of the muscarinic cholinergic agonist oxotremorine (OXO) [76]. They also found, conversely, that ACC lesions blocked the effect of posttraining OXO infusions into the basolateral amygdala [76]. These findings suggest that the ACC and basolateral amygdala may interact in enabling posttraining infusions to enhance memory. It was also demonstrated that the ACC plays a key role for establishing the efficacy and strength of amygdala-dependent auditory fear conditioning, in which excitotoxic lesions and transient inactivation of the ACC in rats selectively caused deficits in the acquisition or expression of amygdala-dependent fear learning [77]. 

In free moving mice, it has been shown that synchronous activity in the ACC and lateral amygdala is necessary for observational fear learning, in which neuronal activities in the ACC were enhanced and synchronized with those of the lateral amygdala at the theta frequency during observational fear [8]. These results suggest that synchronous activities between the ACC and lateral amygdala are necessary for recognition and expression of social fear. Importantly, they indicate that the lateral amygdala is vital for both the acquisition and the retrieval of observational fear, whereas the ACC has a modulatory role in the generation of fear by interacting sensory and affective dimensions. 

A previous study using tracing experiments revealed that the projections between the ACC and basolateral amygdala are glutamatergic [77]. Furthermore, a recent study using an optogenetic technique demonstrated that synaptic plasticity in the ACC-lateral amygdala pathway seems to be under less stringent control by GABA [78]. Therefore, it is likely that activity of ACC during emotional events plays a key role to modulate the amygdala-dependent fear learning. 

It is widely believed that both the ACC and amygdala are involved in pain modulation and emotional responses to pain [4, 79]. A previous report using formalin-induced conditioned place avoidance (F-CPA) and electric foot-shock conditioned place avoidance (S-CPA) suggests that the amygdala mediates both pain- and fear-related negative emotion [80]. More recent studies found that microinjection of ZIP into the ACC could alleviate spontaneous pain [66]. Therefore the ACC plays a critical role in the expression of pain-related negative emotion [80]. However, how does the network between the ACC and amygdala contribute to pain-related emotional responses? Recently, it was reported that pain-related hyperactivity of basolateral amygdala neurons plays important roles in not only emotional-affective aspects of pain but also pain-related decision-making deficits through amygdala-prefrontal cortex circuit [81]. Thus, it is strongly suggested that cognitive impairment is caused by amygdala-driven prefrontal cortical deactivation. Further studies are necessary to understand how the ACC-amygdala pathway contributes to pain-related behavior.

## 10. Future Directions

Remarkable progress has been made in elucidating the molecular and cellular mechanisms underlying the fear learning and memory in the amygdala and ACC. However, our understanding of these mechanisms is not far from complete. Therefore, further studies using integrative methods including neurobiological, neurophysiological, and neuropharmacological approaches are necessary to extend our understanding of these mechanisms. Since fear conditioning is critical as a means of studying brain circuits involved in emotional disorders [82], molecules underlying fear conditioning could be therapeutic targets for treating these disorders. 

It still remains unanswered how the neural network between the ACC and amygdala can be activated during fear acquisition and/or extinction. In order to solve this question, advanced technologies such as *in vivo* multi-electrophysiological methods will be helpful. If multi-electrophysiological recording methods are applied to the ACC and amygdala simultaneously during fear acquisition and/or extinction, functional connectivity between the ACC and amygdala may be elucidated. Future studies are clearly needed to understand how neurotransmitters and neuromodulators affect functional connectivity between the ACC and amygdala during fear learning.
